# Miscellaneous

**Published:** 1858-01

**Authors:** 


### MISCELLANEOUS.

*Cider.*—A pint of mustard-seed put into a barrel of cider will keep it sweet for several months.

*Apples* or *Pears* cut into quarters, stripped of the rind, and the core cut out, baked with a little water and sugar, and eaten with an equal amount of boiled rice, make a delightful and healthful dish for children, for breakfast, or as a dessert for dinner, but not for supper.

*Candle Burning.*—A small piece of candle will give a mild, shady light all night in a sick-room, by putting finely pulverized salt on the candle, until it reaches the black part of the wick.

